# STRATEGIES TO PREVENT FRAILTY: THE POWER OF EARLY DETECTION AND TRAINING OF PROFESSIONALS

**DOI:** 10.1093/geroni/igz038.177

**Published:** 2019-11-08

**Authors:** Nienke Bleijenberg, Niek de Wit

**Affiliations:** 1 University of Applied Sciences Utrecht, Utrecht, Netherlands; 2 Julius Center for Health Sciences and Primary Care, University Medical Center Utrecht, Utrecht, Netherlands

## Abstract

Aging in place is an important goal for both older adults as well as many health policies worldwide, as it is in the Netherlands. Within an aging society, the number of frail older people with complex care needs living at home is increasing. Despite the various definitions of frailty, it is important to early identify who is at risk in clinical practice in order to prevent functional decline, enhance quality of life, and reduce health care costs. Furthermore, an important requirement is effective collaboration between primary care professionals. Various factors are associated with frailty. However, early detection of frailty and its risk factors such as oral health, nutrition and medication related problems is not part of routine care of professionals. To recognize frailty and its risk factors we started a large proactive integrated primary care program that successfully identified frail older people living at home based on routine care data in the Netherlands. After two-year follow-up, a reduction in acute visits at the emergency department was observed. Next, we performed additional studies focusing on early detection and prevention of risk factors of frailty such as oral health, nutrition, and medication related problems among older people living at home. During this symposium we will present the results of the program, followed by our studies that investigated frailty or frailty related risk factors. Additionally, we will show how we enhanced and evaluated the knowledge and skills of professionals working with frail older people in primary care.

